# Hepatoprotective Constituents of Total Dibenzocyclooctadiene Lignans from *Schisandra chinensis* Based on the Spectrum-Effect Relationship

**DOI:** 10.3390/molecules26216554

**Published:** 2021-10-29

**Authors:** Lu-Lu Deng, Xu-Dong Xie, Jiang Li, Dao-Ping Wang, Xiao-Jiang Hao, Gang Chen, Shu-Zhen Mu

**Affiliations:** 1 State Key Laboratory of Functions and Applications of Medicinal Plants, Guizhou Medical University, Guiyang 550014, China; luludeng@gzcnp.cn (L.-L.D.); xiexudongbb@163.com (X.-D.X.); jiangli@gzcnp.cn (J.L.); wdp_7897@aliyun.com (D.-P.W.); haoxj@mail.kib.ac.cn (X.-J.H.); 2 The Key Laboratory of Chemistry for Natural Products of Guizhou Province and Chinese Academy of Sciences/Guizhou Provincial Engineering Research Center for Natural Drugs, Guiyang 550014, China; 3 Shanxi Province Key Laboratory of Environmental Pollution Control and Reservoir Protection Technology of Oilfields, Xi’an Shiyou University, Xi’an 710065, China; gangchen@xsyu.edu.cn

**Keywords:** *Schisandra chinensis*, total dibenzocyclooctadiene lignans (TDL), high-performance liquid chromatography (HPLC) fingerprint, hepatoprotective effects, spectrum-effect relationship, isolation and purification

## Abstract

To scientifically clarify the hepatoprotective constituents of Fructus Schizandrae chinensis, eleven batches samples of total dibenzocyclooctadiene lignans (TDL) from *Schisandra chinensis* were prepared by using the optimum extraction technique. Characteristic high-performance liquid chromatography (HPLC) chromatograms were obtained through HPLC analysis technology, and the hepatoprotective effects of the eleven batches of TDL were evaluated by MTT assay. Based on the chemical and biological activity results, the spectrum-effect relationship between the characteristic HPLC fingerprints and the hepatoprotective effect of TDL was established using Minitab 16.0 data analysis software. On the basis of the spectrum-effect relationship, thirteen compounds (**1**–**13**) were obtained from the TDL by chemical natural product chemical separation and purification technology, and their structures were identified on the basis of the spectral data and the literature. Based on these compounds, thirteen common peaks among the thirty-three chromatographic peaks in the above HPLC fingerprints were identified. Our findings showed that some components, including, schisandrin B (**2**), schisandrin A (**3**), and schisandrol B (**7**) had significant roles in promoting hepatoprotective activity. Preliminary verification of the spectrum-effect relationship of TDL from *S. chinensis* was carried out, and the results confirmed that the activity of a composite of these three key components in optimal ratios was better than that of any individual compound, which potentially confirmed the reliability of the spectrum-effect relationship and the synergistic effects of traditional Chinese medicine.

## 1. Introduction

The dry fruit of *Schisandra chinensis* (Turcz.) Baill. is a basic source plant of Fructus Schisandrae chinensis and is officially listed in the Chinese Pharmacopoeia. As a well-known traditional Chinese medicine (TCM), *S. chinensis* has five flavors, including sour, sweet, bitter, spicy, and salty. Therefore, it is usually called Wuweizi [1]. *S. chinensis* is mainly distributed in Liaoning, Jilin, and Heilongjiang provinces and the North China region. A modern pharmacological study of dibenzocyclooctadiene lignans showed that they had many biological functions, such as liver protection, reduced enzyme levels, antibacterial, antitumour, antioxidant, antiviral effects, and immune enhancement [2,3,4,5,6]. Furthermore, it is noteworthy that *S. chinensis* has been prescribed as a medication for the treatment of liver damage in TCM for thousands of years. In recent years, as a result of chemical and pharmacological research, it was shown that the dibenzocyclooctadiene lignans in *S. chinensis* were the main hepatoprotective constituents [7,8,9,10,11,12], and an enrichment technique for the total dibenzocyclooctadiene lignans (TDL) from *S. chinensis* was reported [13,14]. However, the correlation between the active dibenzocyclooctadiene lignans and their hepatoprotective activities was not clear, especially regarding which lignans are synergistic with respect to the activity and which lignans are less effective.

More and more studies have shown that the efficacy of TCM is due to the comprehensive synergistic effect of multiple specific targets in the material. In recent years, the spectrum-effect relationship has become an important technical means of combining the qualitative basis and curative effect of TCM. TCM fingerprint information can be combined with drug effect information, which has provided new ideas for elucidating the effective mechanism of TCM by screening out characteristic peaks that are closely related to drug efficacy. Therefore, the spectrum-effect relationship of TDL from *S. chinensis* was established to clarify its hepatoprotective effect; the results are of great significance to the development and utilization of Fructus Schisandrae chinensis and provide a new research paradigm for assessing the comprehensive effect of effective substances in TCM.

High-performance liquid chromatography (HPLC) fingerprinting is an important analytical method that is useful in the identification and quality control of botanical medicines [15,16,17,18]. HPLC has undergone such rapid development in the quality control and evaluation in TCM and it can be used to determine the contents and constituents of many compounds to obtain fingerprints of various materials [19,20]. The in vitro CCl_4_-induced hepatoprotective activity model, a classic chemical liver injury bioassay method [21], was chosen to evaluate the hepatoprotective activity of TDL from *S. chinensis*. Minitab, a powerful software for statistical data analysis, was used to describe the contributions of different dibenzocyclooctadiene lignans to hepatoprotective activity.

Herein, HPLC and the in vitro hepatoprotective screening model were used to obtain the characteristic HPLC fingerprint and hepatoprotective activity of TDL from *S. chinensis*. The above data were analyzed in Minitab 16.0 software to obtain the spectrum-effect relationship of TDL from *S. chinensis* and predict an optimal dibenzocyclooctadiene lignan ratio with strong hepatoprotective activity. In our analysis of the chemical constituents of TDL in *S. chinensis*, thirteen compounds with the best hepatoprotective activity were isolated and identified, and thirteen common peaks were identified successfully on this basis. Preliminary verification of the spectrum-effect relationship was carried out, and the results indicated that the activity of three key compounds (schisandrin B, schisandrin A, and schisandrol B) in the optimal ratio was better than that of any individual compound. These results might provide an experimental basis and new research strategies for the research and development of *S. chinensis* as a hepatoprotective drug and health care product.

## 2. Results

### 2.1. Establishing the Typical HPLC Chromatogram of TDL from S. chinensis 

**Figure 1:**
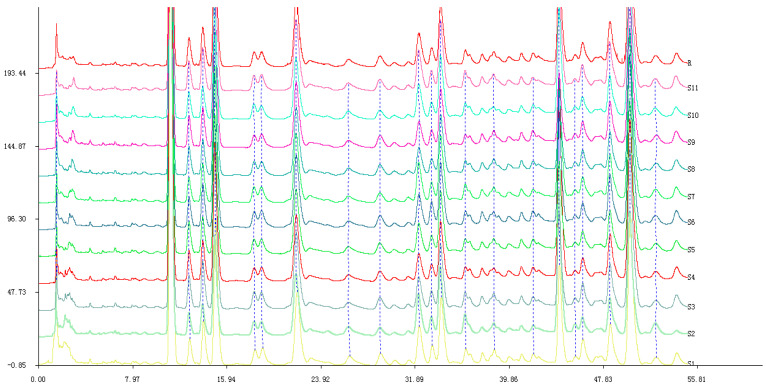
The characteristic HPLC chromatogram of TDL from *S. chinensis*.

**Figure 2:**
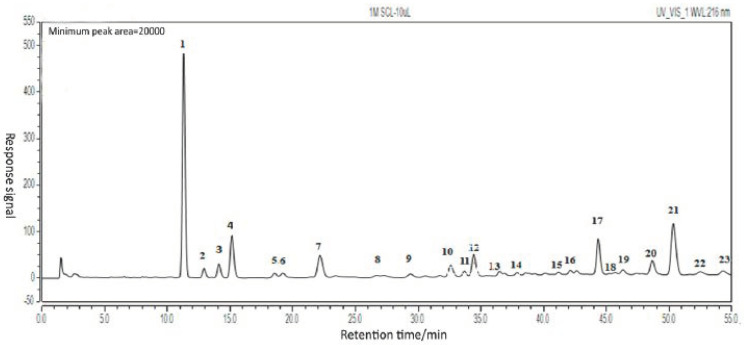
The common peaks of characteristic HPLC chromatogram of TDL from *S. chinensis*.

The details optimized extraction technology of TDL are shown in the Supplementary Materials (Tables S1–S5). Samples **S1**–**S11** were analyzed by HPLC. The typical HPLC chromatogram of TDL from *S. chinensis* is shown in Figure 1. The HPLC chromatogram of **S11** is shown in Figure 2. Twenty-three common peaks with large areas and good segregation from adjacent peaks were identified the eleven HPLC chromatograms, and the total areas of these peaks were more than 80% of the total peak area in every chromatogram. Therefore, these twenty-three peaks were selected as the common characteristic peaks, and they were numbered as shown in Figure 2. The data in Table 1 show the average peak areas and relative retention times of the twenty-three common characteristic peaks of **S1**–**S11**.

The experimental results showed that the relative standard deviation (RSD, %) values for the relative retention times from different batches of samples were less than 0.2%, which showed that the relative retention time of the same chromatographic peak was very consistent. Thus, using this method to establish the feature maps was scientific and reasonable. The RSD (%) values for many peak areas were greater than 10%, which indicated that the compounds in different batches of samples were similar, but their contents were not identical, which implied that the method was suitable for analyzing the spectrum-effect relationship.

### 2.2. Biological Activity

#### 2.2.1. Protective Activity of Different Concentrations of Test Samples against CCl_4_-Induced Liver Injury

The protective activity of three doses of **S11** against CCl_4_-induced liver injury was measured by the MTT method, and the OD values and average cell survival rates (%) are shown in Table 2. Compared with the blank group, the cell survival rate of the model group was 68.87%, which indicated that the experimental model had been constructed successfully. The cell survival rates of the positive control (dimethyl dicarboxylate biphenyl, DDB) group and the three experimental groups were higher than those of the model group, which showed that the samples had a certain protective effect against injury to liver cells. The cell survival rate of the middle-dose group was much higher than that of the DDB group, which showed that this dose had better protective activity against CCl_4_-induced liver injury.

#### 2.2.2. Protective Activity of Eleven Test Samples against CCl_4_-Induced Liver Injury

The protective activities of **S1**–**S11** against CCl_4_-induced liver injury were screened by the MTT method, and the OD values and average cell survival rates (%) are presented in Table 3. The results showed that these eleven samples of *S. chinensis* had a certain protective effect against CCl_4_-induced liver injury in HL-7702 cells. The protective effects of **S1**, **S4**, **S6**, and **S11** were the most significant.

### 2.3. Isolation of TDL from S. chinensis

**Figure 3:**
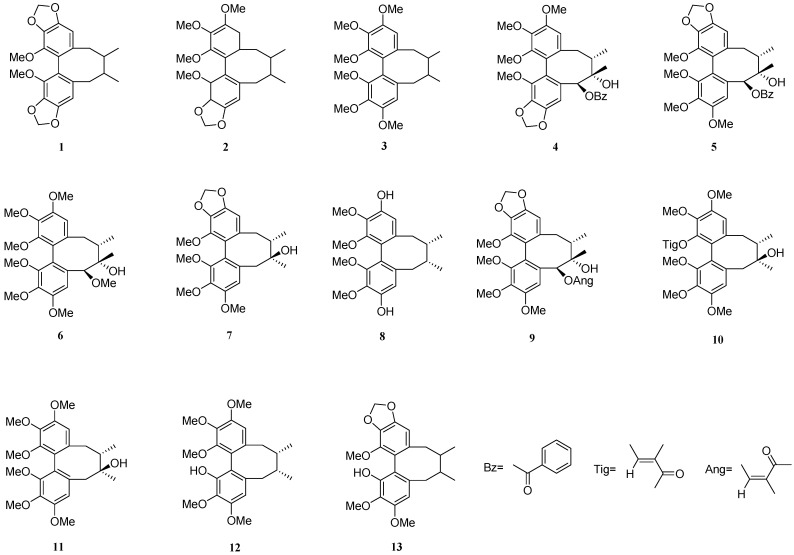
Structures of compounds **1**–**13** from total dibenzocyclooctadiene lignans of *S. chinensis*.

Thirteen compounds (**1**–**13**) were isolated and purified from TDL from *S. chinensis* by silica gel column chromatography, reversed phase silica gel column chromatography, Sephadex LX-20 gel column chromatography, semi-prepared HPLC, and recrystallized. Based on the obtained NMR, MS data, and the literatures reported, their structures were identified as schisandrin C (**1**) [22], schisandrin B (**2**) [23], schisandrin A (**3**) [24], gomisin G (**4**) [25], schisantherin A (**5**) [26], angeloylgomisin Q (**6**) [27], schisandrol B (**7**) [27], gomisin J (**8**) [28,29], schisantherin B (**9**) [30], tigloylgomisin H (**10**) [31], schisandrin (**11**) [24], schisanhenol (**12**) [32], and schisanhenol B (**13**) [33] (Figure 3). All the ^1^H and ^13^C NMR spectra data for compounds **1**–**13** are presented in Supplementary Materials.

### 2.4. Evaluation of Similarity and Assignment of Common Peaks

The Similarity Evaluation System for Chromatographic Fingerprints of Traditional Chinese Medicines was used to evaluate the similarity of the HPLC chromatograms of the eleven Fructus Schisandrae chinensis samples, and the results are shown in Table 4. The similarity index of these eleven samples (**S1**–**S11**) was higher than 0.97.

**Figure 4:**
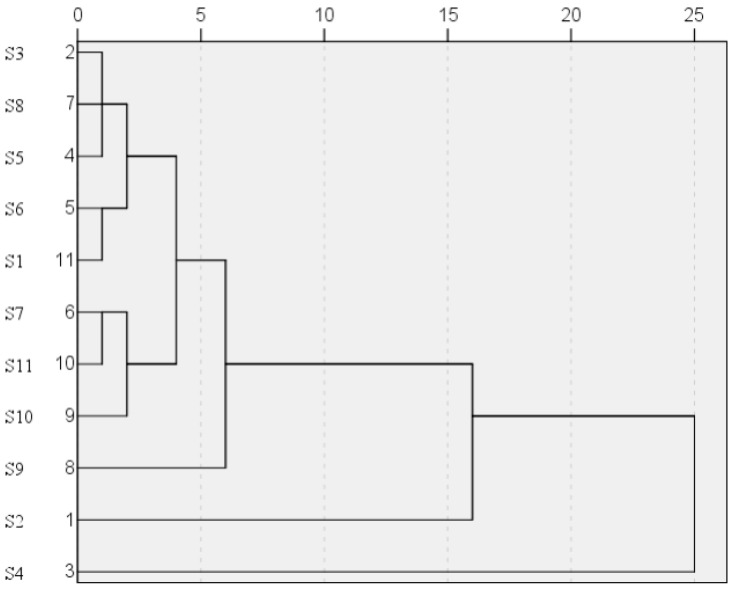
Hierarchical cluster analysis of *S. chinensis*.

Hierarchical cluster analysis is a method for highlighting the similarities and differences with the common peak area data of these eleven batches of *S. chinensis.* Taking the peak areas of twenty-three common peaks as the variable, the systematic cluster analysis of samples **S1**–**S11** was carried out. The average connection method between groups was adopted, and the cosine of the included angle was used as the sample similarity measure. As shown in Figure 4, the samples could be divided into two main categories. The one category included **S1**–**S3**, and **S5**–**S11**, and the other category only included **S4**. These results indicated the little difference of the content and distribution of chemical compounds in various *S. chinensis*.

**Figure 5:**
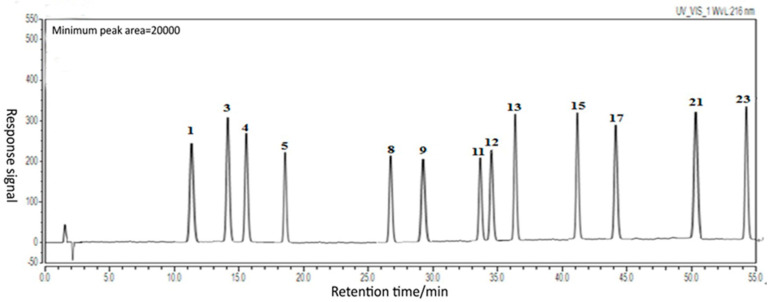
The HPLC chromatogram of mixed reference substances.

Thirteen compounds and their mixture were analyzed by HPLC under the same chromatographic conditions to determine their retention times, and the HPLC chromatogram of the mixed reference substances is shown in Figure 5. They were identified according to their retention times. According to Section 2.3 and the relevant literature, thirteen peaks were confirmed, and their retention times, corresponding compound names, and molecular weights are presented in Table 5.

### 2.5. Analysis of the Spectrum-Effect Relationship

A spectrum-effect relationship model between the protective effect of the eleven samples and the characteristic HPLC fingerprints was established.

The standardized regression coefficients were calculated by the partial least squares regression model. According to the standardized regression coefficients in Table 6, the regression equation of the twenty-three common peak areas and the hepatoprotective activity was obtained as the following: Y = − 0.288X_1_ − 0.309X_2_ − 0.064X_3_ + 0.275X_4_ + 0.104X_5_ − 0.254X_6_ − 0.580X_7_ − 0.012X_8_ + 0.588X_9_ + 0.067X_10_ + 0.525X_11_ + 0.378X_12_ + 0.086X_13_ + 0.49X_14_ + 0.204X_15_ − 0.206X_16_ + 0.347X_17_ − 0.394X_18_ + 0.281X_19_ − 0.008X_20_ + 0.705X_21_ + 0.028X_22_ − 0.375X_23_.

As shown in Table 6, thirteen chromatographic peaks corresponding to specific compounds were directly proportional to the hepatoprotective effect, which indicated that these thirteen compounds could promote the hepatoprotective effect. The peak areas of these thirteen compounds are listed in Table 1. Ten peaks were inversely proportional to the hepatoprotective effect. The strength of the hepatoprotective effects of these compounds was in the following order: peak **21** > peak **9** > peak **11** > peak **14** > peak **12** > peak **17** > peak **19** > peak **4** > peak **15** > peak **5** > peak **13** > peak **10** > peak **22** > peak **20** > peak **8** > peak **3** > peak **16** > peak **6** > peak **1** > peak **2** > peak **23** > peak **18** > peak **7**.

According to the pharmacological activity screening results shown in Table 3, the protective activity of **S4** was the greatest. **S4** was taken as an example; according to Table 5 and Table 6, among the thirteen effective compounds, nine compounds were identified, including schisandrin B (**2**), schisandrin A (**3**), gomisin G (**4**), schisantherin A (**5**), schisandrol B (**7**), schisantherin B (**9**), tigloylgomisin H (**10**), schisanhenol (**12**), and schisanhenol B (**13**). Among these nine compounds, seven compounds play obvious roles in protection against liver injury, and the peak area ratio was schisandrin B (**2**):gomisin G (**4**):schisantherin A (**5**):schisantherin B (**9**):schisandrin A (**3**):schisandrol B (**7**):schisanhenol B (**13**) = 17:1:1:5:16:11:1. And the other ten compounds have inhibitory effects on liver protective activities.

### 2.6. Preliminary Validation of Hepatoprotective Effect of Three Key Compounds In Vitro

On the basis of the above spectrum-effect relationship, three key compounds schisandrin B (**2**), schisandrin A (**3**), and schisandrol B (**7**), were selected for the validation experiment. The protective activities of composites containing these three compounds in various proportions against CCl_4_-induced liver injury were screened by MTT, and the OD values and average cell survival rates (%) are presented in Table 7. The results showed that the activity of these three compounds combined according to the selected ratio was better than that of any individual compound, which was consistent with the result of the spectrum-effect relationship.

## 3. Discussion

Chinese herbal medicine, with the advantage of high efficiency, low toxicity, and less side effects, has become an important resource for the modern drug development. *S. Chinensis* is a plant species well-known in TCM, and also in modern Chinese medicine, with hepatoprotective activity and reduces related serum enzymes [34]. However, due to the multitarget and multicomponent characteristics of TCM, it is difficult to control the quality of TCM and it has become a hot topic in the field of research. Recently, many studies have proved that the spectrum-effect relationship should be an effective method to control the quality of TCM because it can connect chromatographic fingerprints with biological activity to explore the quality markers related to clinical efficacy [35]. Thus, to ensure the quality and therapeutic consistency of TCM, this approach may be a good strategy to control its quality.

This study examined eleven batches of *S. chinensis* samples, on the basis of the preliminary experimental results, their TDL (**S1**–**S11**) were extracted and enriched as putative effective components under the optimum conditions. A characteristic HPLC fingerprint was established. The protective activities of eleven TDL samples (**S1**–**S11**) were obtained using the CCl_4_-induced liver injury model and the MTT method. Then, using Minitab 16.0 data analysis software, the relationship between chemical constituents and pharmacological activities was analyzed, and the related equations were obtained. The separation and structural identification of thirteen compounds (**1**–**13**) from TDL in *S. chinensis* was used to identify the most common peaks. The results showed that schisandrin B (**2**), schisandrin A (**3**), gomisin G (**4**), schisantherin A (**5**), schisandrol B (**7**), schisantherin B (**9**) and schisanhenol B (**13**) played obvious roles in protection against liver injury, and the preliminary optimal proportions were schisandrin B (**2**):gomisin G (**4**):schisantherin A (**5**):schisantherin B (**9**):schisandrin A (**3**):schisandrol B (**7**):schisanhenol B (**13**) = 17:1:1:5:16:11:1. On the basis of the above spectrum-effect relationship, three key compounds schisandrin B (**2**), schisandrin A (**3**), and schisandrol B (**7**), were selected for the preliminary validation experiment. The results showed that the activity of the three key compounds combined in the optimal ratio was better than that of any individual compound, which was consistent with the result of the spectrum-effect relationship.

In our study, the spectrum-effect relationship between the chromatographic fingerprints of TDL and the hepatoprotective activity in *S. chinensis* was established for the first time, and the main effective components and their best ratio were obtained. Our findings will provide a more scientific and reasonable method for improving the quality control standard of *S. chinensis*, and it is of great significance for explaining the hepatoprotective activity of TDL and developing new hepatoprotective drugs. In addition, in this work, thirteen common peaks were identified. Compared with the results of current literature, our results were improved, which provided a basis for expounding the material basis of TDL. Due to the limitation of experimental conditions and time, only thirteen compounds were obtained, and activity tests were conducted preliminarily in vitro. Next, in order to further improve the quality control system of *S. chinensis*, more compounds will be prepared, and to explore more active constituents or metabolites, mass spectrometry analysis will be considered for our research.

## 4. Materials and Methods

### 4.1. Chemicals and Equipment

The following materials and reagents were used in separation, purification, and HPLC analysis: silica gel G and H (Qingdao Ocean Chemical Plant Branch, Qingdao, China), reversed-phase silica gel (Rp-18, 40–63 μm) (Amersham Biosciences, Uppsala, Sweden), octadecyl silane (ODS, 50 µm) (Merck; Darmstadt, Germany), and Sephadex LH-20 (Amersham Biosciences, Uppsala, Sweden) for column chromatography. TLC analyses were carried out using GF254 TLC plates (Qingdao Puke Separation Material Co., Ltd., Qingdao, China). All solvents used for the preparation of the extracts were analytical-grade reagents and were purchased from Tianjin Kemiou Chemical Reagent Co. (Tianjin, China) Acetonitrile (HPLC grade) and methanol (HPLC grade) were purchased from Sigma Chemical Co. (Budapest, Hungary), and ultrapure water was prepared with a Milli-Q water purification system (Millipore, Bedford, USA).

NMR spectra were recorded on an INOVA-400 and 600 MHz superconducting nuclear magnetic resonance spectrometer (TMS internal standard, Varian, USA) with deuterated reagents for NMR spectroscopy (Wuhan Spectrum Company of Chinese Academy of Sciences, Wuhan, China). HPLC analysis was performed with a DIONEX U3000 HPLC system (Diane China Co., LTD., Beijing, China quaternionic pump, DAD detector, Chromeleon 7.2 chromatography workstation). The common peaks MS data were analyzed by a HPMS5973 mass spectrometer (HP, Agilent, Palo Alto, USA).

The following materials were also used in the experiments: normal liver cell line HL-7702 (National Collection of Authenticated Cell Cultures, Beijing, China); fetal bovine serum (Gibco, USA); dimethyl sulfoxide (DMSO) and 3-(4,5-dimethyl-2-thiazolyl)-2,5-diphenyl-2-H-tetrazolium bromide (MTT) (Beijing Mr. Lai Treasure Company, Beijing, China); phosphate buffer solution (PBS), RPMI (Roswell Park Memorial Institute) 1640 medium, penicillin, streptomycin and 0.25% trypsin solution (containing phenolred) (HyClone); CCl4 (analytical reagent, AR) (Sinopharm Chemical Reagent Co., Ltd., Beijing, China); and cell culture bottles, centrifuge tubes, 96 cell culture plates and other consumables (NEST Biotechnology Co. Ltd., Wuxi, China). The medicine diphenyl dimethylester (DDB) was used as a positive control. The absorbance was read using a microplate reader (Varioskan LUX, Thermo, Waltham, USA) at 490 nm. The results were obtained in at least three independent experiments.

### 4.2. Plant Material

Samples of dried fruit of *S. chinensis* were collected from several regions of China between August and October 2014 (as shown in Table 8). Botanical identifications were performed by Professor Lisha Dong from Guiyang University of Chinese Medicine, and voucher specimens for each sample material (Nos. 20140801-20140811) were deposited at the Herbarium of the Key Laboratory of Chemistry for Natural Products of Guizhou Province and the Chinese Academy of Sciences.

### 4.3. Preparation of Total Dibenzocyclooctadiene Lignans (TDL)

TDL samples from eleven different batches of dried *S. chinensis* were prepared separately (S1–S11). Each batch of dried *S. chinensis* (10.0 g) was crushed, sieved to 40 mesh and then refluxed with a solid-liquid ratio (g/mL) of 1:7 in 90% ethanol (2 × 4 h). After filtration, the solution was evaporated to dryness under reduced pressure. The residue was dissolved in water and extracted 5 times with 6 volumes of ethyl acetate, the ethyl acetate layers were combined, and the solvent was reduced to obtain the ethyl acetate extract of each batch of *S. chinensis*. The above ethyl acetate extract dissolved in ethanol was adsorbed with an AB-8 macroporous resin (Shanghai Science and Technology Development Co., Shanghai, China) at a ratio of 1:10 and then eluted with an ethanol-water series (6 volumes of 30% ethanol and 20 volumes of 95% ethanol). Finally, the constituents eluted with 95% ethanol were collected as the TDL (S1–S11). Then, each batch of TDL (10.0 mg) was accurately weighed, dissolved in methanol, and brought to volume with methanol in a 10 mL volumetric flask. Then, 1.5 mL of the sample solution was filtered with a 0.45 μm microporous membrane for HPLC analysis.

The reference substances used for HPLC analysis were isolated by us, dissolved in HPLC-grade methanol at a concentration of 0.04 mg/mL, and then filtered with a 0.45 μm microporous membrane for HPLC analysis. Aliquots (100 μL) of each reference substance were taken and mixed to obtain the mixed standard.

### 4.4. Typical HPLC Chromatograms of Different TDL Samples from Eleven Batches of S. chinensis

Eleven different batches of TDL (**S1**–**S11**) were analyzed by HPLC using a DIONEX U3000 HPLC system. The chromatographic conditions were as follows: solution A: acetonitrile; solution B: water; mobile phase: A 50% from 0 to 25 min, A 65% from 25 to 35 min, A 70% from 35 to 40 min, and finally A 70% from 40 to 55 min. The wavelength was 216 nm, the sample volume was 10 μL, the column temperature was 25°C, and the flow rate was 1.0 mL/min. Separation was performed using a Kromasil 100-5 C18 column from AKZO NOBEL (particle size: 5 μm, length×diameter: 250 × 4.6 mm).

### 4.5. Hepatoprotective Activity of Different TDL Samples from Eleven Batches of S. chinensis against CCl_4_-Induced Liver Injury

The cell line was cultured as described in the relevant literature [36]. To assess cell viability, 1.0 × 10^4^ cells were plated in each well of 96-well plates, and the plates were placed in a humidified 5% CO_2_ incubator at 37 °C for 24 h. Solutions of the test samples (S1–S11) (25 μL, 50 μg/mL) were added to the 96-well plates and incubated for 24 h. Next, 4 μL of CCl_4_ was added, and incubation was continued for 12 h. After 20 μL of MTT was added to each well and incubated for 4 h, the old culture medium was discarded. Next, 150 μL of DMSO was added to each well, and the plates were oscillated for 15 min at 37°C to ensure that the crystals were dissolved completely. Finally, the absorbances (OD values) were analyzed using an enzyme-linked instrument at 490 nm to evaluate the hepatoprotective effects of the TDL (**S1**–**S11**) from eleven different batches of *S. chinensis* against CCl_4_-induced liver injury. Fourteen experimental groups, including a blank group, a CCl_4_ model group, a positive control group and the sample groups, were used in this bioassay. Each group was tested in five parallel wells and repeated three times. The cell survival rate (%) was calculated relative to that of the normal group.

### 4.6. Isolation of TDL with the Best Hepatoprotective Activity

Sample S4 had the best hepatoprotective activity, so it was selected for separation and purification. As described in Section 4.3, 400 g TDL from sample **S4** was obtained and mixed with 40–80 mesh silica gel at a ratio of 1:1.2. The mixed sample was separated by 7 rounds of elution through a silica gel (200–300 mesh) column with a gradient of petroleum ether:ethyl acetate (volume ratios was 30:1, 8:1, 2:1, and 1:1) to yield 6 fractions (Fr. **A**–**E**). Then, the different fractions were separated by silica gel column chromatography, Sephadex LX-20 gel column chromatography, semi-prepared HPLC, and repeated recrystallization.

Fr. **A** was first subjected to normal-pressure silica gel chromatography (200–300 mesh, petroleum ether:ethyl acetate = 40:1). Then, the mixture was purified again with a 300–400 mesh normal-pressure silica gel column (petroleum ether-ethyl acetate = 80:1) to give compound **1** (schisandrin C, 684 mg). Fr. **B** was separated by silica gel chromatography (200–300 mesh, petroleum ether-ethyl acetate = 40:1), and recrystallized repeatedly to obtain compound **2** (schisandrin B, 8.412 g) and compound **3** (schisandrin A, 4.801 g)

Fr. **C** was subjected to silica gel chromatography (200–300 mesh, petroleum ether:acetone = 20:1) to afford 12 subfractions (Fr. **C.1**–**12**), and recrystallized repeatedly to obtain compound **4** (gomisin G, 321 mg) from Fr. **C.12**. Then, compound **5** (schisantherin A, 20 mg) was further obtained by passing the sample through two silica gel columns (300–400 mesh, chloroform:acetone = 80:1 and chloroform-methanol = 100:0–0:100 respectively). Then, the sample was purified by silica gel column (300–400 mesh, chloroform:acetone = 30:1) and semipreparative HPLC (MeOH:H_2_O = 68:32, v = 1.5 mL/min ) to give compound **6** (angeloylgomisin Q, 51 mg). Fr.C.10 was separated by silica gel chromatography (300–400 mesh, chloroform:acetone = 60:1), and by recrystallization to yield compound **7** (schisandrol B, 333 mg). Further purification by semi-prepared HPLC (MeOH:H_2_O = 70:30, v = 1.0 mL/min) afforded compound **8** (gomisin J, 13 mg) and compound **9** (schisantherin B, 9 mg).

Fr. **D** was separated by a silica gel (200–300 mesh, petroleum ether:acetone = 8:1). Compound **10** (tigloylgomisin H, 51 mg) was prepared by repeated silica gel column, Sephadex LH-20 column, and semi-prepared HPLC. After further purification with medium-pressure ODS (MeOH:H_2_O = 40:100, 60:100, 80:100 and 90:100), compound **11** (schsandrin, 20 g) was finally obtained by repeated recrystallized.

Fr. **E** was subjected to silica gel chromatography (200–300 mesh, petroleum ether-ethyl acetate:formic acid = 15:5:1) to afford 5 subfractions (Fr. **E.1**–**5**). Fr. **E.2** and Fr. **E.4** were separated by repeated silica gel column and Sephadex LH-20 column. Compound **12** (schisanhenol, 32 mg) was obtained by vacuum column chromatography (silica H, petroleum ether:acetone = 5:1), and compound **13** (schisanhenol B, 15 mg) was obtained by purification with an Rp-18 column (actone:H_2_O = 40:60–70:30).

The structures of compounds **1**–**13** were identified by ^1^H-NMR, ^13^C-NMR, MS, and comparison with reported data.

### 4.7. Spectrum-Effect Relationship

#### 4.7.1. Similarity Analysis and Hierarchical Cluster Analysis

Similarity analysis was performed by the Similarity Evaluation System for Chromatographic Fingerprints of Traditional Chinese Medicines (Version 2021A; Beijing, China). Hierarchical cluster analysis of the eleven samples **S1**–**S11** was performed using SPSS software (version 24).

#### 4.7.2. Partial Least Squares Regression Analysis

The twenty-three common chromatographic peak areas were set as the independent variable (X), their hepatoprotective effect were taken as dependent variables (Y), and the regression models were built sequentially. After the principal components were extracted, the linear relationships were exhibited by the partial least squares regression model. Meanwhile, the regression coefficient was revealed and considered as the index to exhibit the relative impact of the predictor variables on the response variable for this model. The partial least squares regression was performed using Mini tab 16.0 statistics software.

### 4.8. Preliminary Verification Test of the Spectrum-Effect Relationship

Similar to the experimental method in Section 4.5 above, we selected three compounds, schisandrin A, schisandrin B, and schisandrol B, that have a great influence on the spectrum-effect relationship for the validation experiment. Ten experimental groups, including a blank group, a CCl_4_ model group, a positive control group and the sample groups, were used in this bioassay. Each group was tested in five parallel wells and repeated three times. The cell survival rate (%) was calculated relative to that of the normal group.

## 5. Conclusions

This work established HPLC fingerprints using eleven batches of TDL from *S. chinensis*. The similarity and hierarchical cluster analysis more objectively explain the characteristic of the source of raw materials. In addition, the relationship between the characteristic HPLC fingerprints and the hepatoprotective effect of TDL were analyzed by partial least squares regression method, and according to the thirteen common peaks identified, the main effective components and their optimal ratios were clarified, and the mixture of three key active components schisandrin B (**2**), schisandrin A (**3**), and schisandrol B (**7**) in optimal ratio were screened in vitro. The results, based on above analytical techniques, showed that these three compounds were consistent with the spectrum-effect relationship on hepatoprotective effect. This report provided a scientific and rational experimental basis for perfecting the quality control standards for *S. chinensis* and provides important guidance for explaining the hepatoprotective effect of TDL from Fructus Schisandrae chinensis and developing hepatoprotective medicines or health products.

## Figures and Tables

**Table 1:** The average peak area and relative retention time of the twenty-three common characteristic peaks.

Peaks	Retention Time/min	Areas										
		S1	S2	S3	S4	S5	S6	S7	S8	S9	S10	S11
**1**	11.15	124.2	121.9	135.0	118.1	125.4	130.2	130.3	119.4	111.0	122.6	128.0
**2**	12.74	5.744	5.342	7.530	6.814	7.629	6.449	5.492	5.943	7.148	6.381	6.959
**3**	13.90	10.74	7.499	11.34	9.607	9.081	10.64	9.876	8.222	9.315	8.159	10.49
**4**	14.92	36.63	25.68	39.93	34.71	35.23	38.52	34.68	35.66	38.06	29.82	35.92
**5**	18.26	4.229	3.960	4.923	3.849	4.206	4.586	4.387	3.932	2.015	4.350	4.338
**6**	18.88	6.182	4.999	5.929	5.018	5.544	6.479	5.722	5.062	6.635	5.047	5.995
**7**	21.82	24.22	20.23	25.55	21.63	21.59	23.77	23.38	22.62	21.54	22.67	25.60
**8**	26.25	5.475	5.042	4.611	4.109	4.620	4.668	4.218	4.543	4.614	4.856	4.916
**9**	28.91	4.404	2.958	4.360	3.756	4.132	4.427	3.661	3.908	4.528	3.449	4.268
**10**	32.20	11.75	13.03	9.745	8.090	8.972	9.536	12.08	8.055	7.510	10.83	12.23
**11**	33.28	3.373	3.306	3.176	3.860	3.095	4.650	3.396	2.845	3.095	2.763	3.369
**12**	34.04	20.32	20.75	19.87	17.15	19.74	20.54	21.17	18.76	18.43	19.90	21.60
**13**	36.14	3.382	6.233	3.445	2.544	2.803	2.934	5.433	2.547	2.228	2.899	4.366
**14**	38.54	6.299	3.393	6.570	5.476	5.720	4.367	4.150	6.293	6.458	5.053	6.923
**15**	40.89	3.001	3.000	3.478	3.158	3.150	3.171	3.329	3.514	3.608	2.860	3.638
**16**	41.88	2.868	2.763	2.967	2.831	2.933	3.303	2.900	3.268	3.882	2.692	3.193
**17**	44.08	26.28	37.22	25.05	50.42	23.63	28.98	32.96	21.79	19.10	29.04	32.65
**18**	45.43	2.820	2.043	3.245	2.049	2.211	2.405	3.224	3.519	3.725	2.269	3.418
**19**	46.07	8.192	4.637	7.945	5.808	5.470	5.768	7.591	7.295	7.520	5.262	8.023
**20**	48.38	13.44	11.48	14.34	13.08	12.21	15.03	15.27	12.95	13.97	12.30	15.67
**21**	50.06	51.70	41.98	53.91	53.89	51.53	56.58	55.74	48.19	49.75	49.74	55.27
**22**	52.26	2.848	4.435	2.857	2.721	2.861	3.134	3.639	2.954	2.922	2.979	3.686
**23**	54.01	4.317	3.000	4.807	4.154	3.709	4.820	4.121	3.912	4.466	3.523	4.828

**Table 2:** The protective activity of different concentrations of sample S11 against CCl_4_-induced liver injury ($\bar{x}$ ± *s*, n = 3).

Group	The OD Value	Cell Survival Rate/%
Blank	0.615 ± 0.024	100.00 ± 0.00
Model	0.424 ± 0.030	68.87 ± 4.88
DDB ^a^	0.446 ± 0.091	72.54 ± 14.81
**S11**–1 (0.01 mg/mL)	0.437 ± 0.012	71.08 ± 3.91
**S11**–2 (0.05 mg/mL)	0.466 ± 0.026	75.76 ± 4.23
**S11**–3 (0.10 mg/mL)	0.439 ± 0.022	71.30 ± 3.58

^a^ DDB is the positive group.

**Table 3:** The protective activity of the eleven batches test samples against CCl_4_-induced liver injury ($\bar{x}$ ± *s*, n = 3).

Group	The OD Value	Cell Survival Rate/%
Blank	0.696 ± 0.035	100.00 ± 0.00
Model	0.430 ± 0.024	61.80 ± 3.45
DDB	0.460 ± 0.022	66.06 ± 3.16
**S1**	0.473 ± 0.022	68.01 ± 3.16
**S2**	0.438 ± 0.026	62.93 ± 3.74
**S3**	0.441 ± 0.180	63.34 ± 11.49
**S4**	0.493 ± 0.023	70.83 ± 3.30
**S5**	0.460 ± 0.018	66.03 ± 2.59
**S6**	0.481 ± 0.082	69.11 ± 11.78
**S7**	0.470 ± 0.027	67.56 ± 3.88
**S8**	0.440 ± 0.129	63.27 ± 18.53
**S9**	0.436 ± 0.035	62.59 ± 5.03
**S10**	0.432 ± 0.035	62.07 ± 5.03
**S11**	0.473 ± 0.026	67.93 ± 3.74

Note: The concentration of DDB and samples **S1–S11** was 0.05 mg/mL.

**Table 4:** The similarity of the eleven batches of TDL samples.

	S1	S2	S3	S4	S5	S6	S7	S8	S9	S10	S11	Reference
**S1**	1	0.989	0.998	0.983	0.998	0.998	0.997	0.998	0.997	0.997	0.998	0.998
**S2**	0.989	1	0.987	0.987	0.989	0.989	0.993	0.985	0.977	0.993	0.991	0.991
**S3**	0.998	0.987	1	0.98	0.999	0.998	0.996	0.999	0.996	0.996	0.997	0.998
**S4**	0.983	0.987	0.98	1	0.982	0.987	0.988	0.979	0.976	0.987	0.988	0.988
**S5**	0.998	0.989	0.999	0.982	1	0.999	0.996	0.998	0.996	0.998	0.996	0.999
**S6**	0.998	0.989	0.998	0.987	0.999	1	0.998	0.997	0.996	0.998	0.998	0.999
**S7**	0.997	0.993	0.996	0.988	0.996	0.998	1	0.996	0.993	0.998	0.999	0.998
**S8**	0.998	0.985	0.999	0.979	0.998	0.997	0.996	1	0.998	0.997	0.997	0.998
**S9**	0.997	0.977	0.996	0.976	0.996	0.996	0.993	0.998	1	0.993	0.994	0.995
**S10**	0.997	0.993	0.996	0.987	0.998	0.998	0.998	0.997	0.993	1	0.998	0.999
**S11**	0.998	0.991	0.997	0.988	0.996	0.998	0.999	0.997	0.994	0.998	1	0.998
Reference	0.998	0.991	0.998	0.988	0.999	0.999	0.998	0.998	0.995	0.999	0.998	1

**Table 5:** The identified common peaks of characteristic chromatogram.

Peaks	Compound	Retention Time/min	Molecular Weight	Relative Peak Areas/%
**1**	schisandrin (**11**)	11.287	432	30.25
**3**	gomisin J (**8**)	14.103	388	2.25
**4**	schisandrol B (**7**)	15.130	416	7.51
**5**	tigloylgomisin H (**10**)	18.543	500	0.99
**8**	angeloylgomisin Q (**6**)	26.690	530	0.68
**9**	gomisin G (**4**)	29.367	536	0.97
**11**	schisantherin A (**5**)	33.667	536	0.90
**12**	schisantherin B (**9**)	34.413	514	4.57
**13**	schisanhenol (**12**)	36.477	402	1.29
**15**	schisanhenol B (**13**)	41.170	386	0.89
**17**	schisandrin A (**3**)	44.340	416	7.01
**21**	schisandrin B (**2**)	50.333	400	12.14
**23**	schisandrin C (**1**)	54.297	384	1.10

**Table 6:** Correlation coefficients of common peaks of partial least squares analysis.

Peaks	Coefficients	Peaks	Coefficients	Peaks	Coefficients
**1**	−0.288	**9**	0.588	**17**	0.347
**2**	−0.309	**10**	0.067	**18**	−0.394
**3**	−0.064	**11**	0.525	**19**	0.281
**4**	0.275	**12**	0.378	**20**	−0.008
**5**	0.104	**13**	0.086	**21**	0.705
**6**	−0.254	**14**	0.490	**22**	0.028
**7**	−0.580	**15**	0.204	**23**	−0.375
**8**	−0.012	**16**	−0.206		

**Table 7:** The protective activity of schisandrin B (**2**), schisandrin A (**3**) and schisandrol B (**7**) against CCl_4_-induced liver injury ($\bar{x}$ ± *s*, n = 3).

Group	The OD Value	Cell Survival Rate/%
Blank	0.562 ± 0.019	100.00 ± 0.00
Model	0.263 ± 0.067	47.57 ± 4.37
DDB	0.332 ± 0.051	55.72 ± 4.71
Combination 1	0.365 ± 0.063	61.32 ± 6.97
Combination 2	0.217 ± 0.060	35.44 ± 7.24
Combination 3	0.390 ± 0.063	64.36 ± 6.85
Combination 4	0.129 ± 0.039	21.44 ± 4.68
schisandrin B (**2**)	0.096 ± 0.044	15.78 ± 5.99
schisandrin A (**3**)	0.114 ± 0.043	18.89 ± 5.63
schisandrol B (**7**)	0.139 ± 0.054	22.99 ± 7.12

Note: “Combination 1” is schisandrin B (**2**):schisandrin A (**3**):schisandrol B (**7**) = 17:16:11, “Combination 2” is schisandrin B (**2**):schisandrin A (**3**) = 17:16, “Combination 3” is schisandrin B (**2**):schisandrol B (**7**) = 17:11, “Combination 4” is schisandrin A (**3**):schisandrol B (**7**) = 16:11.

**Table 8:** Sample information.

Sample	Origin	Sample	Origin
**S1**	Heilongjiang, China	**S7**	Hebei 2, China
**S2**	Shanxi, China	**S8**	Shaanxi, China
**S3**	Inner Mongolia, China	**S9**	Ningxia, China
**S4**	Liaoning, China	**S10**	Shandong, China
**S5**	Jilin, China	**S11**	Gansu, China
**S6**	Hebei 1, China		

## Data Availability

The data presented in this study are available in the supplementary materials.

## Supplementary Materials

The following are available online. The ^1^H NMR and ^13^C NMR spectral data for compounds **1**–**13**, and the optimized extraction technique for TDL from *Schisandra chinensis* fruit. Table S1: The data of single factor experiments, Table S2: The factors and levels table of L_9_ (3^4^) orthogonal experiment, Table S3: The orthogonal experiment table, Table S4: The results of orthogonal test, Table S5: The analysis of variance for orthogonal test.

## Sample Availability

Samples of the compounds **1**–**13** are available from the authors.
