# Supplementary material for: Hepatoprotective Constituents of Total Dibenzocyclooctadiene Lignans from Schisandra chinensis Based on the Spectrum-Effect Relationship
Source: Molecules. 2021 Oct 29;26(21):6554. doi: 10.3390/molecules26216554 (PMC8587461; doi:10.3390/molecules26216554)
Supplement: Supplementary file 1 [file molecules-26-06554-s001.zip › molecules-1416761-supplementary.pdf]

Supporting Information

Hepatoprotective constituents of total dibenzocyclooctadiene lignans from *Schisandra chinensis* based on the spectrum-effect relationship

Lu-Lu Deng [1,2], Xu-Dong Xie [1, 2], Jiang Li [1, 2], Dao-Ping Wang [1, 2], Xiao-Jiang Hao [1,2], Gang Chen [3], Shu-Zhen Mu [1,2,*]

[1] State Key Laboratory of Functions and Applications of Medicinal Plants, Guizhou Medical University, Guiyang 550014, China; luludeng@gzcnp.cn (L.-L.D.); xiexudongbb@163.com (X.-D.X.); jiangli@gzcnp.cn (J.L.); wdp_7897@aliyun.com (D.-P.W.); haoxj@mail.kib.ac.cn (X.-J.H.)

[2] The Key Laboratory of Chemistry for Natural Products of Guizhou Province and Chinese Academy of Sciences/Guizhou Provincial Engineering Research Center for Natural Drugs, Guiyang 550014, China

[3] Shanxi Province Key Laboratory of Environmental Pollution Control and Reservoir Protection Technology of Oilfields, Xi'an Shiyou University, Xi'an 710065, China; gangchen@xsyu.edu.cn (G.C.)

* Correspondence: muzi0558@126.com

^{1}H and ^{13}C NMR spectra data for compounds S1−S13

Schisandrin C (**S1**): Colorless plates; ESI-MS: *m/z* 385 $[M + H]^+$, 791 $[2M + Na]^+$; ^{1}H NMR (400 MHz, $CDCl_3$) δ: 6.48 (2H, d, *J* = 1.9 Hz, H-4, H-11), 5.97-5.90 (4H, m, 2×-OCH_2O-), 3.82 (6H, d, *J* = 5.3 Hz, 2× -OCH_3), 2.58-1.96 (4H, m, H-6, H-9), 1.92-1.70 (2H, m, H-7, H-8), 0.96 (3H, d, *J* = 7.2 Hz, 8-CH_3), 0.73 (3H, d, *J* = 7.1 Hz, 7-CH_3); ^{13}C NMR (100 MHz, $CDCl_3$) δ: 141.2 (C-1) , 134.3 (C-2), 148.6 (C-3), 103.1 (C-4), 138.2 (C-5), 35.5 (C-6), 40.7 (C-7), 33.6 (C-8), 38.7 (C-9), 132.7 (C-10), 106.1 (C-11), 147.7 (C-12), 134.6 (C-13), 140.9 (C-14), 122.1 (C-15), 120.9 (C-16), 12.5 (C-CH_3), 21.7 (C-CH_3), 100.7 (C-OCH_2O-), 59.60 (2×C-OCH_3).

Schisandrin B (**S2**): White crystal (Et_2O/hexane); ESI-MS: *m/z* 401 $[M + H]^+$, 823 $[2M + Na]^+$; ^{1}H NMR (400 MHz, $CDCl_3$) δ: 6.55 (1H, s, H-4), 6.47 (1H, s, H-11), 5.94 (2H, d, *J* = 0.9 Hz, -OCH_2O-), 3.89 (6H, d, *J* = 4.7 Hz, 2×-OCH_3), 3.81 (3H, s, -OCH_3), 3.54 (3H, s, -OCH_3), 2.64-1.96 (4H, m, H-6, H-9), 1.96-1.71 (2H, m, H-7, H-8), 0.97 (3H, d, *J* = 7.2 Hz, 8-CH_3), 0.73 (3H, d, *J* = 7.1 Hz, 7-CH_3); ^{13}C NMR (100 MHz, $CDCl_3$) δ: 141.0 (C-1), 134.5 (C-2), 148.6 (C-3), 110.5 (C-4), 137.8 (C-5), 35.4 (C-6), 40.6 (C-7), 33.5 (C-8), 39.1 (C-9), 134.0 (C-10), 102.9 (C-11), 151.6 (C-12), 140.0 (C-13), 151.5 (C-14), 123.2 (C-15), 121.6 (C-16), 12.7 (C-CH_3), 21.8 (C-CH_3), 100.7 (C-OCH_2O-), 61.0 (C-OCH_3), 60.5 (C-OCH_3), 59.6 (C-OCH_3), 55.8 (C-OCH_3).

Schisandrin A (**S3**): White powder; ESI-MS: *m/z* 417 $[M + H]^+$, 855 $[2M + Na]^+$; ^{1}H NMR (400 MHz, $CDCl_3$) δ: 6.53 (2H, d, *J* = 3.0 Hz, H-4, H-11), 3.88 (12H, dd, *J* = 5.6, 4.4 Hz, 4×-OCH_3), 3.58 (6H, d, *J* = 1.1 Hz, 2×-OCH_3), 2.64-2.00 (4H, m, H-6, H-9), 1.97-1.72 (2H, m, H-7, H-8), 1.00 (3H, d, *J* = 7.1 Hz, 7-CH_3), 0.74 (3H, d, *J* = 7.1 Hz,
8-CH_3).

Gomisin G (**S4**): Colorless transparent solid; ESI-MS: *m/z* 559 $[M + Na]^+$, 1095 $[2M + Na]^+$; ^{1}H NMR (400 MHz, $CDCl_3$) δ: 6.78 (1H, s, H-11), 6.69 (1H, s, H-4),

6.01

(2H, dd, J = 9.6, 1.3 Hz, -OCH_2O-), 5.88 (1H, s, H-6), 3.98 (3H, s, -OCH_3), 3.82 (3H, s, -OCH_3), 3.39 (3H, s, -OCH_3), 3.15 (3H, s, -OCH_3), 2.54-2.26 (2H, m, H-9), 1.67 (1H, s, 7-OH), 1.51 (1H, s, H-8), 1.34 (3H, s, 7-CH_3), 1.21 (3H, d, J = 7.1 Hz, 8-CH_3), -OBz: 7.46 (1H, t, -OBz-H), 7.37 (2H, d, J = 7.1 Hz, -OBz-H), 7.29-7.23 (2H, m, -OBz-H); ^{13}C NMR (100 MHz, $CDCl_3$) δ: 150.6 (C-1), 139.8 (C-2), 148.0 (C-3), 106.1 (C-4), 136.5 (C-5), 84.3 (C-6), 72.4 (C-7), 42.1 (C-8), 36.7 (C-9), 139.8 (C-10), 107.2 (C-11), 153.2 (C-12), 141.6 (C-13), 150.7 (C-1), 121.7 (C-15), 122.2 (C-16), 18.9 (C-CH_3), 28.2 (C-CH_3), 101.4 (C-OCH_2O-), 59.7 (C-OCH_3), 59.8 (C-OCH_3), 60.1 (C-OCH_3), 56.0 (C-OCH_3), 164.7 (C-1'), 133.2 (C-2'), 129.4 (C-3'), 128.7 (C-4'), 137.1 (C-5'), 128.1 (C-6'), 129.2 (C-7').

Schisantherin A (**S5**): Colourless prisms; ESI-MS: *m/z* 559 [M + Na] $^+$, 1095 [2M + Na]$^+$; ^{1}H NMR (400 MHz, $CDCl_3$) δ: 6.82 (1H, s, H-4), 6.56 (1H, s, H-11), 5.82 (1H, s, H-6), 5.77 (1H, d, J = 1.1 Hz, -OCH_2O-), 5.64 (1H, d, J = 1.1 Hz, -OCH_2O-), 3.92 (3H, s, -OCH_3), 3.88 (3H, s, -OCH_3), 3.56 (3H, s, -OCH_3), 3.30 (3H, s, -OCH_3), 2.22-2.35 (2H, dt, J = 48.9, 11.9 Hz, H-9), 2.17-2.05 (1H, m, H-8), 1.55 (1H, s, 7-OH), 1.37 (3H, s, 7-CH_3), 1.18 (3H, d, J = 7.1 Hz, 8-CH_3), -OBz: 7.49-7.31 (3H, m, J = 18.1 Hz, 7.4 Hz, -OBz-H), 7.31 (2H, t, J = 7.7 Hz, -OBz-H); ^{13}C NMR (100 MHz, $CDCl_3$) δ: 140.1 (C-1), 134.1 (C-2), 148.7 (C-3), 102.4 (C-4), 135.0 (C-5), 84.8 (C-6), 42.6 (C-8), 36.4 (C-9), 130.2 (C-10), 109.8 (C-11), 151.9 (C-12), 141.7 (C-13), 152.2 (C-14), 120.9 (C-15), 122.1 (C-16), 18.9 (C-CH_3), 28.1 (C-CH_3), 60.8 (C-OCH_3), 60.7 (C-OCH_3), 60.5 (C-OCH_3), 58.6 (C-OCH_3), 55.9 (C-OCH_3), 164.7(C-1'), 132.9 (C-2'), 129.5 (C-3'), 129.3 (C-4'), 129.3 (C-5'), 127.8 (C-6'), 127.8 (C-7').

Angeloylgomisin Q (**S6**): Yellow oil; ESI-MS:*m/z* 553 [M + Na$^+$], 1083 [2M + Na$^+$];

^{1}H NMR (400 MHz, $CDCl_3$) δ: 6.79 (1H, s, H-4), 6.54 (1H, s, H-8), 5.73 (1H, s, H-6), 3.91-3.87 (9H, m, 3×-OCH_3), 3.82 (3H, s, -OCH_3), 3.57 (3H, s, -OCH_3), 3.51 (3H,

s, $-OCH_3$), 2.39-2.17 (2H, m, H-9), 2.03-1.94 (1H, m, 7-OH), 1.63-1.45 (1H, m, H-8), 1.30 (3H, s, 7-CH_3), 1.19-1.14 (3H, m, 7-CH_3), -OAng: 6.01-5.91 (1H, m, -OAng-H), 1.84-1.78 (3H, m, -OAng-b-CH_3), 1.33 (3H, s, -OAng-a-CH_3); ^{13}C NMR (125 MHz, $CDCl_3$) δ: 150.8 (C-1), 141.9 (C-2), 153.2 (C-3), 107.3 (C-4), 126.9 (C-5), 84.3 (C-6), 72.4 (C-7), 42.2 (C-8), 36.8 (C-9), 139.9 (C-10), 110.2 (C-11), 152.2 (C-12), 140.9 (C-13), 151.9 (C-14), 122.3 (C-15), 122.54 (C-16), 15.7 (C-17), 19.8 (C-18), 56.2 (C-19), 60.9 (C-OCH_3), 60.7 (C-OCH_3), 60.6 (C-OCH_3), 60.3 (C-OCH_3), 55.9 (C-OCH_3), 165.9 (C-1'), 130.4 (C-2'), 136.6 (C-3'), 19.8 (C-4'), 19.2(C-5').

Schisandrol B (**S7**): White solid; ESI-MS: *m/z* 417 $[M + H]^+$, 855 $[2M + Na]^+$; ^{1}H NMR (400 MHz, $CDCl_3$) δ: 6.61 (1H, s, H-11), 6.47 (1H, s, H-4), 5.95 (2H, dd, J = 4.5, 1.2 Hz, $-OCH_2O-$), 3.89 (6H, s, 2×$-OCH_3$), 3.83 (3H, s, $-OCH_3$), 3.51 (3H, s, -OCH_3), 2.71-2.27 (4H, m, H-6, H-9), 1.92 (1H, s, 8-OH), 1.85 (1H, t, J = 7.2 Hz, H-7), 1.24 (3H, s, 8-CH_3), 0.81 (3H, d, J = 7.3 Hz, 7-CH_3), ^{13}C NMR (100 MHz, $CDCl_3$) δ: 152.0 (C-1), 140.5 (C-2), 152.1 (C-3), 110.2 (C-4), 131.9 (C-5), 71.6 (C-6), 40.3 (C-7), 41.8 (C-8), 33.5 (C-9), 132.3 (C-10), 105.8 (C-11), 147.7 (C-12), 134.8 (C-13), 141.0 (C-14), 121.7 (C-15), 124.0 (C-16), 100.7 (C-OCH_2O-), 15.7 (C-CH_3), 29.9 (C-CH_3), 60.9 (C-OCH_3), 60.5 (C-OCH_3), 59.5 (C-OCH_3), 55.8 (C-OCH_3).

Gomisin J (**S8**): Colorless oil; ESI-MS: *m/z* 389 $[M + H]^+$, 799 $[2M + Na]^+$; ^{1}H NMR (400 MHz, $CDCl_3$) δ: 6.62 (2H, d, H-4, H-11), 5.75 (2H, d, 3-OH, 12-OH), 3.92 (6H, d, J = 3.3 Hz, 2×$-OCH_3$), 3.52 (6H, d, J = 0.6Hz, 2×$-OCH_3$), 2.22-2.19 (4H, m, H-6, H-9), 1.75-1.94 (2H, m, H-7, H-8), 0.97 (3H, d, J = 7.1 Hz, $-CH_3$), 0.72 (3H, d, J = 7.1 Hz, $-CH_3$); ^{13}C NMR (100 MHz, $CDCl_3$) δ: 148.6 (C-1), 140.2 (C-2), 150.3 (C-3), 110.0 (C-4), 137.3 (C-5), 38.8 (C-6), 40.9 (C-7), 40.9 (C-8), 38.8 (C-9), 134.8 (C-10), 109.9 (C-11), 150.2 (C-12), 137.6 (C-13), 147.5 (C-14), 121.4 (C-15), 122.4 (C-16), 21.7 (C-CH_3), 12.5 (C-CH_3), 60.9 (C-OCH_3), 60.0 (C-OCH_3), 60.0 (C-OCH_3), 60.9

(C-OCH_3).

Schisantherin B (**S9**): Yellow crystal (acetone); ESI-MS: *m/z* 537 $[M + Na]^+$, 1051 $[2M + Na]^+$; ^1H NMR (400 MHz, $CDCl_3$) δ: 6.76 (1H, s, H-11), 6.45 (1H, s, H-4), 5.88 (2H, dd, J = 11.4, 1.3 Hz, -OCH_2O-), 5.59 (1H, s, H-6), 3.89 (6H, t, J = 4.1 Hz, 2×-OCH_3), 3.72 (3H, s, -OCH_3), 3.55 (3H, s, -OCH_3), 2.28-1.94 (2H, m, H-9), 1.42-1.35 (1H, m, 7-OH), 1.33 (3H, s, H-7), 1.12 (3H, d, J = 7.1 Hz, H-8), -OAng: 5.99 (1H, m, -OAng-H), 1.84 (3H, dd, J = 7.3, 1.5 Hz, -OAng-b-CH3), 1.60 (3H, s, -OAng-a-CH_3); ^{13}C NMR (100 MHz, $CDCl_3$): δ: 140.2 (C-1), 133.9 (C-2), 148.4 (C-3), 102.4 (C-4), 135.1 (C-5), 36.1 (C-6), 42.2 (C-7), 71.9 (C-8), 84.1 (C-9), 130.7 (C-10), 110.0 (C-11), 151.6 (C-12), 141.3 (C-13), 151.6 (C-14), 121.0 (C-15), 121.8 (C-16), 18.7 (C-CH_3), 28.0 (C-CH_3), 58.7 (C-OCH_3), 60.3 (C-OCH_3), 60.5 (C-OCH_3), 55.3 (C-OCH_3), 100.3 (C-OCH_2O-), 19.5, 15.5 (C-CH_3), 126.8 (C), 139.8 (C), 165.9 (C=O).

Tigloylgomisin H (**S10**): Yellow powder; ESI-MS: *m/z* 501 $[M + Na]^+$, 1023 $[2M + Na]^+$; ^1H NMR (400 MHz, $CDCl_3$) δ: 6.68 (1H, s, H-11), 6.55 (1H, s, H-4), 3.90 (3H, s, -OCH_3), 3.87 (3H, s, -OCH_3), 3.83 (6H, d, J = 4.9 Hz, 2×-OCH_3), 3.50 (3H, s, -OCH_3), 2.75-2.28 (4H, m, H-6, H-9), 1.97-1.85 (1H, m, H-8), 1.61 (1H, s, 7-OH), 1.24 (3H, s, 7-CH_3), 0.85 (3H, d, J = 7.2 Hz, 8-CH_3), -OTig: 6.82 (1H, dd, J = 7.2, 1.7 Hz, -OTig-H), 1.70 (6H, d, J = 5.5 Hz, -OTig-CH_3); ^{13}C NMR (100 MHz, $CDCl_3$) δ: 151.6 (C-1), 142.5 (C-2), 151.7 (C-3), 110.1 (C-4), 132.9 (C-5), 40.5 (C-6), 71.8 (C-7), 41.9 (C-8), 34.1 (C-9), 133.6 (C-10), 112.6 (C-11), 152.3 (C-12), 139.7 (C-13), 140.2 (C-14), 122.8 (C-15), 123.1 (C-16), 15.8 (C-CH_3), 29.8 (C-CH_3), 60.9 (C-OCH_3), 60.8 (C-OCH_3), 60.6 (C-OCH_3), 60.0 (C-OCH_3), 55.9 (C-OCH_3),127.8 (C-1'), 138.2 (C-2'), 14.3 (C-3'), 12.1 (C-4').

Schisandrin (**S11**): Colorless crystal (EtOH); ESI-MS: *m/z* 432 $[M + H]^+$, 887 $[2M + Na]^+$; ^1H NMR (400 MHz, $CDCl_3$) δ: 6.61 (1H, s, H-11), 6.54 (1H, s, H-4), 3.89 (12H,

dd, J = 6.5, 4.5 Hz, -OCH_3), 3.58 (16H, d, J = 4.2 Hz, -OCH_3), 2.70-2.32 (4H, m, H-6, H-9), 1.89 (1H, s, 7-OH), 1.63 (1H, s, H-8), 1.25 (3H, s, 8-CH_3), 0.82 (3H, d, J = 7.2 Hz, 7-CH_3); ^{13}C NMR (100 MHz, $CDCl_3$) δ: 151.7 (C-1), 140.6 (C-2), 152.3 (C-3), 110.3 (C-4), 131.8 (C-5), 40.7 (C-6), 71.7 (C-7), 41.7 (C-8), 34.1 (C-9), 133.8 (C-10), 109.9 (C-11), 151.9 (C-12), 140.0 (C-13), 151.4 (C-14), 122.6 (C-15), 124.1 (C-16), 15.8 (C-CH_3), 29.7 (C-CH_3), 60.9 (C-OCH_3), 60.9 (C-OCH_3), 60.5 (C-OCH_3), 60.5 (C-OCH_3), 55.8 (C-OCH_3), 55.8 (C-OCH_3).

Schisanhenol (**S12**): Colorless crystal (acetone); EI-MS*m/z*: 402 [M[$^+$; ^{1}H NMR (400 MHz, $CDCl_3$) δ: 6.58 (1H, s, H-4), 6.38 (1H, s, H-11), 3.88 and 3.92 (12H, s, 4×-OCH_3), 0.77 (3H, d, J = 6.8Hz, H-7), 1.02 (3H, d, J = 6.8Hz, H-8), 5.76 (1H, s, -OH); ^{13}C NMR (100 MHz, $CDCl_3$) δ: 151.1 (C-1), 139.8 (C-2), 153.0 (C-3), 107.1 (,C-4), 139.6 (C-5), 35.6 (C-6), 40.7 (C-7), 33.6 (C-8), 39.1 (C-9), 134.2 (C-10), 107.6 (C-11), 150.4 (C-12), 133.6 (C-13), 146.7 (C-14), 116.8 (C-15), 121.0 (C-16), 12.6 (C-CH_3), 21.8 (C-CH_3), 61.0 (C-OCH_3), 60.9 (C-OCH_3), 60.9 (C-OCH_3), 55.8 (C-OCH_3), 55.7 (C-OCH_3).

Schisanhenol B (**S13**): Colorless powder; EI-MS *m/z*: 402 [M[$^+$; ^{1}H-NMR (600 MHz, $CDCl_3$) δ: 6.51 (1H, s, H-11), 6.38 (1H, s, H-4), 5.96 (1H, d, J = 1.4 Hz, OCH_2O), 5.93 (1H, d, J = 1.4 Hz， OCH_2O), 5.70 (1H, brs, OH), 3.91 (3H, s, 3-MeO), 3.88 (3H, s, 2-MeO), 3.86 (3H, s, 14-MeO), 1.89 (1H, m，H-8), 1.78 (1H, m，H-7), 0.97 (3H, d, J = 7.4 Hz，H-17), 0.74 (3H, d, J = 7.4 Hz，H-18)； ^{13}C-NMR (150 MHz, CDCl3) δ: 12.9（C-CH_3）, 21.5（C-CH_3）, 33.5（C-7）, 35.7（C-9）, 39.2（C-6）, 40.8（C-8）, 55.7 (C-OCH_3）, 59.8 (C-OCH_3）, 61.0（C-OCH_3）, 100.8 (-OCH_2O）, 103.5（C-11）, 107.2（C-4）, 116.6 (C-16）, 120.2（C-15）, 133.5（C-2）, 134.5（C-5）, 134.7（C-10）, 138.4（C-13）, 141.0（C-14）, 146.9（C-1）, 148.9（C-12）, 150.4（C-3）.

The optimized extraction technology of the total dibenzocyclooctadiene lignans from *Schisandra chinensis*

The optimized extraction technology of the total dibenzocyclooctadiene lignans from *Schisandra chinensis* was determined according to the single factor experiment and orthogonal experiment. And the results showed that the optimized extraction technology is extracting 2 times with 90% ethanol (4 h, 4 h), and the solid-liquid ratio of 1:7. The detailed methods and results of single factor and orthogonal experiment were as follows.

1. The single factor experiment

a. The determination of ethanol concentration

The dried fruit of *Schisandrae chinensis* was crushed and sieved to 40 mesh, weighed five samples (each sample was 10.0 g), and then refluxed with 55%, 65%, 75%, 85% and 95% ethanol (50 mL) for 3 h, respectively. After concentrating, the extracts were dissolved adequately in 50 mL 80% methanol, and took 1 mL supernatant into a 10 mL volumetric flask, and diluted to 10 mL with 80% methanol. Then, 1.5 mL of the sample solution was filtered with 0.45 μm microporous membrane for HPLC analysis. These five samples were numbered as A-55, A-65, A-75, A-85 and A-95, respectively.

The analysis of these five samples was carried out on a Kromasil 100-5 C18 column (particle size: 5 μm, length×diameter: 250 × 4.6 mm). The mobile phase were methanol-water series, and eluted with 70% methanol. The wavelength was 220 nm, the flow rate was 1.5 mL/min, the sample volume was 10 μL, and the column temperature was kept at 30°C. The concentrations of three dibenzocyclooctadiene

lignans, schisandrin A, schisandrin B and schisandrin were calculated according to the peak areas and standard curve. This experiment was repeated 3 times, and the results were averaged. According to comparing the concentrations of these three compounds, the optimum concentration of ethanol was determined.

b. The determination of extraction time

The dried fruit of *Schisandrae chinensis* was crushed and sieved to 40 mesh, weighed five samples (each sample was 10.0 g), and then refluxed with 50 mL 85% ethanol for 1, 2, 3, 4 and 5 h, respectively. After concentrating, the extracts were dissolved adequately in 50 mL 80% methanol, and took 1 mL supernatant into a 10 mL volumetric flask, and diluted to 10 mL with 80% methanol. Then, 1.5 mL of the sample solution was filtered with 0.45 μm microporous membrane for HPLC analysis. These five samples were numbered as t-1, t-2, t-3, t-4 and t-5, respectively.

The chromatographic conditions were the same as above. This experiment was repeated 3 times, and the results were averaged. According to comparing the concentrations of these three compounds, the optimum extraction time was determined.

c. The determination of extraction times

The dried fruit of *Schisandrae chinensis* was crushed and sieved to 40 mesh, weighed five samples (each sample was 10.0 g), and then refluxed with 50 mL 85% ethanol (1, 2, 3, 4 and 5×3 h). After concentrating, the extracts were dissolved adequately in 50 mL 80% methanol, and took 1 mL supernatant into a 10 mL volumetric flask, and diluted to 10 mL with 80% methanol. Then, 1.5 mL of the

sample solution was filtered with 0.45 μm microporous membrane for HPLC analysis. These five samples were numbered as N-1, N-2, N-3, N-4 and N-5, respectively.

The chromatographic conditions were the same as above. This experiment was repeated 3 times, and the results were averaged. According to comparing the concentrations of these three compounds, the optimum extraction times was determined.

d. The determination of solid-liquid ratio

The dried fruit of *Schisandrae chinensis* was crushed and sieved to 40 mesh, weighed five samples (each sample was 10.0 g), and then refluxed with a solid-liquid ratio (g/mL) of 1:2, 1:3, 1:4, 1:5 and 1:6 in 50 mL 85% ethanol (3 h). After concentrating, the extracts were dissolved adequately in 50 mL 80% methanol, and took 1 mL supernatant into a 10 mL volumetric flask, and diluted to 10 mL with 80% methanol. Then, 1.5 mL of the sample solution was filtered with 0.45 μm microporous membrane for HPLC analysis. These five samples were numbered as L-1, L-2, L-3, L-4 and L-5, respectively.

The chromatographic conditions were the same as above. This experiment was repeated 3 times, and the results were averaged. According to comparing the concentrations of these three compounds, the optimum extraction solid-liquid ratio was determined.

According to the single factor experiments, five levels were selected, the ethanol concentration, extraction time, extraction times, and solid-liquid ratio. And the concentrations of schisandrin A, schisandrin B and schisandrin were analysed by

HPLC, the higher contcentration meant the better extraction condition. The experimental results are shown in Table S1. When the concentration of ethanol was 95%, the concentrations of the three reference materials achieved the highest. With the increase of extraction time, the concentration of schisandrin is the highest when the extraction time is 4 h, and the concentrations of schisandrin A and schisandrin B are similar when the extraction time is 5 h, so the optimum extraction time is chosen to be 4 h. With the increase of extraction times, the concentration of these three reference materials in extract reduce sharply. When the sample is extracted 3 times, the concentrations of schisandrin A, schisandrin B and schisandrin are very low. Considering the cost and time, the optimal extraction times is determined to be 2 times. With the increase of extraction solvent amount, the concentrations of three compounds are on the rise, so the optimum solid-liquid ratio is determined to be 1:6.

Table S1. The data of single factor experiments (n=3)

Group	extract/g	Peak areas/A			concentration/mg·g^{-1}		
		A*	B*	C*	A	B	C
A-55	2.27	80.6990	13.8237	14.2930	4.6660	0.7586	0.7980
A-65	2.53	81.8674	15.2072	22.2776	4.7335	0.8338	1.2381
A-75	2.37	85.6914	20.0238	29.5285	4.9544	1.0957	1.6378
A-85	2.33	84.9471	21.4722	33.6411	4.9114	1.1745	1.8645
A-95	2.49	86.2868	22.5401	37.3064	4.9888	1.2326	2.0665
t-1	2.16	78.0531	19.7979	30.3024	4.5132	1.0835	1.6804
t-2	2.17	73.3433	18.8920	29.0622	4.2411	1.0342	1.6121
t-3	2.34	75.2710	19.2401	30.0882	4.3525	1.0531	1.6686
t-4	2.37	85.0810	20.2691	33.1181	4.9192	1.1091	1.8356
t-5	2.30	84.1389	20.8622	33.3670	4.8647	1.1413	1.8494
N-1	2.33	75.2710	19.2401	30.0882	4.3525	1.0531	1.6686
N-2	0.70	19.6183	5.4611	11.7502	1.1375	0.3039	0.6578
N-3	0.21	3.6545	0.9681	3.1792	0.2153	0.0596	0.1854
N-4	0.09	1.1362	0.2294	0.0000	0.0698	0.0194	0.0101
N-5	0.05	0.3208	0.0000	0.0000	0.0227	0.0069	0.0101

L-2	1.59	63.9701	15.2601	23.0834	3.6996	0.8367	1.2825
L-3	1.86	71.7573	17.7575	26.2630	4.1495	0.9725	1.4578
L-4	2.10	76.5751	19.2401	28.8578	4.4278	1.0531	1.6008
L-5	2.27	75.2710	19.2401	30.0882	4.3525	1.0531	1.6686
L-6	2.47	86.3425	21.5067	34.8894	4.9920	1.1764	1.9333

Note: A* schisandrin, **B*** schisandrin A, **C***schisandrin B.

2. The orthogonal experiment

According to the results of single factor experiments, four factors are set, the ethanol concentration (A), extraction time (B), extraction times (C), and the solid-liquid ratio (D), three levels are set in each factor, the factors and levels of L_9 (3^4) orthogonal experiment are shown in Table S2. Using the SPSS 20.0 software, the experiment scheme of orthogonal experiment are designed, which are shown in Table 3.

The dried fruit of *Schisandrae chinensis* was crushed and sieved to 40 mesh, weighed nine samples (each sample was 10.0 g), and then refluxed according to the extraction techniques in the Table S3, respectively. After concentrating, the extracts were dissolved adequately in 50 mL 80% methanol, and took 1 mL supernatant into a 10 mL volumetric flask, and diluted to 10 mL with 80% methanol. Then, 1.5 mL of the sample solution was filtered with 0.45 μm microporous membrane for HPLC analysis. The chromatographic conditions were the same as above. This experiment was repeated 5 times, and the results were averaged. According to comparing the concentrations of above three compounds, the optimum extraction technology was determined.

Table S2. The factors and levels table of L_9 (3^4) orthogonal experiment

Level	Factor			
	A/%	B/h	C/times	D
1	85	3	1	1:5
2	90	4	2	1:6
3	95	5	3	1:7

Table S3. The orthogonal experiment table

Group	Factor			
	A/%	B/h	C/times	D
1	A_1	B_1	C_1	D_1
2	A_1	B_2	C_2	D_2
3	A_1	B_3	C_3	D_3
4	A_2	B_1	C_2	D_3
5	A_2	B_2	C_3	D_1
6	A_2	B_3	C_1	D_2
7	A_3	B_1	C_3	D_2
8	A_3	B_2	C_1	D_3
9	A_3	B_3	C_2	D_1

The experimental results are shown in Table S4. According to the range analysis, the primary and secondary factors which impacted the concentrations of schisandrin A, schisandrin B and schisandrin are known as: C > B > D > A. When schisandrin is chosen as an index, the optimum extraction technology is $A_2B_2C_2D_2$, and when schisandrin A or schisandrin B is chosen as the index, the optimum extraction technology is $A_2B_2C_2D_3$.

Table S4. The results of orthogonal test (n=5)

Group	Factor				Index components		
	A/%	B/h	C/times	D	schisandrin /mg·g^{-1}	schisandrin A /mg·g^{-1}	schisandrin B /mg·g^{-1}
1	A_1	B_1	C_1	D_1	5.243	1.234	2.002
2	A_1	B_2	C_2	D_2	6.359	1.569	2.677
3	A_1	B_3	C_3	D_3	5.911	1.450	2.525
4	A_2	B_1	C_2	D_3	6.281	1.567	2.734
5	A_2	B_2	C_3	D_1	6.086	1.511	2.664
6	A_2	B_3	C_1	D_2	5.508	1.329	2.179

7		A_3	B_1	C_3	D_2	6.038	1.440	2.574
8		A_3	B_2	C_1	D_3	5.679	1.404	2.356
9		A_3	B_3	C_2	D_1	5.872	1.471	2.494
schisandrin /mg·g^{-1}	K1	5.838	5.854	5.477	5.734	$R_C>R_B>R_D>R_A$		
	K2	5.958	6.041	6.171	5.968			
	K3	5.863	5.764	6.012	5.957			
	R	0.120	0.280	0.690	0.240			
schisandrin A /mg·g^{-1}	K1	1.418	1.414	1.322	1.405	$R_C>R_B>R_D>R_A$		
	K2	1.469	1.495	1.536	1.446			
	K3	1.438	1.416	1.467	1.474			
	R	0.051	0.081	0.214	0.069			
schisandrin B /mg·g^{-1}	K1	2.401	2.437	2.179	2.387	$R_C>R_B>R_D>R_A$		
	K2	2.526	2.566	2.635	2.477			
	K3	2.475	2.399	2.588	2.538			
	R	0.125	0.166	0.456	0.152			

The variance analysis result of orthogonal test is shown in Table S5. The significance of each factor was analysed by the method of variance analysis. When schisandrin was chosen as the index ingredient, the *P* value of factor A was less than 0.10, this indicated that factor A had a certain influence on the concentration of schisandrin, the *P* value of factor B or D was both less than 0.05, this explained that factor B and D had a significant effect on the concentration of schisandrin, the *P* value of factor C was less than 0.01, this showed factor C was very significant for the content of schisandrin. According to the result of variance analysis, when schisandrin A and schisandrin B were chosen as the index components, the results were similar to that. And the result of variance analysis was consistent with that of the range analysis. Considering the K_2 and K_3 value of schisandrin was similar, eventually the optimum extraction technology of total dibenzocyclooctadiene lignans was determined to be

$A_2B_2C_2D_3$, which was the following: refluxing with 90% ethanol (2×4 h), and the solid-liquid ratio was 1:7.

Table S5. The analysis of variance for orthogonal test

**Index components**	**Factors**	**SS_j**	**Degree of freedom (f)**	**Mean square**	**F values**	***P* values**
schisandrin	A	0.024	2	0.012	1	< 0.10
	B	0.120	2	0.06	4.932	< 0.05
	C	0.793	2	0.397	32.592	< 0.01
	D	0.105	2	0.052	4.303	< 0.05
schisandrin A	A	0.004	2	0.002	1	< 0.10
	B	0.013	2	0.006	3.185	< 0.05
	C	0.071	2	0.036	17.9	< 0.01
	D	0.007	2	0.004	1.791	< 0.05
schisandrin B	A	0.024	2	0.012	1	< 0.10
	B	0.046	2	0.023	1.944	< 0.05
	C	0.377	2	0.189	16.03	< 0.01
	D	0.035	2	0.017	1.482	< 0.05
